# Supplementary material for: Tussocks facilitate their neighbours mainly by ameliorating extreme temperatures in tropical high mountains
Source: PLoS One. 2020 Nov 13;15(11):e0242313. doi: 10.1371/journal.pone.0242313 (PMC7665801; doi:10.1371/journal.pone.0242313)
Supplement: Attachment — Submitted filename: Response to Reviewers.docx [file pone.0242313.s003.docx]

**RESPONSE TO REVIEWER COMMENTS**

**(Line numbers correspond to the document without change control)**

**REVIEWER 1**

**Comment 1.** I don’t know why the authors only use two traits (plant longevity and plant size) to represent plant survival and growth under different treatments? Does plant size in the text mean plant diameter? There is no detail information in the M&M section. The effects of different pressures on plant growth are shown in different aspects, such as plant height, crown width, root growth, leaf traits, biomass, root/shoot ratio. Choosing the suitable traits to present the plant performance is very important in the study.

*Answer:*

Yes, the influence of different stressors can be reflected on several plant traits. Our experimental design only considered the effect of stressors on two traits: plant size, measured as plant diameter, and plant longevity. Because we only measure two traits, our conclusions may be limited. Yet, we have two reasons for only measuring plant size and longevity. The first is to do with the plant we studied. *Mexerion* is a small rosette that occurs mainly appressed to the ground in the early stages of its life. This makes measuring plant height and root/shoot ratio difficult. We choose to measure plant diameter because it was an easy trait to measure. The second reason is to do with the experiment itself. Because we were also tracking plant longevity during the experiment, we did not want to measure a property during the experiment that could, in any way, have a negative effect on plants. As such, we did not want to unbury plants to measure root growth. We agree that measuring other properties that reflect plant performance would have strengthened our findings. Yet, we had to compromise given the constraints imposed by the studied plant and the experimental design, and thus only measured plant size and longevity. We have expanded the reasoning of our experimental design, it now read as (L185-190): ‘Though many plant traits can be used as surrogates for performance (e.g. plant height, root growth or leaf traits), we chose to only measure plant size and longevity because of two constraints. First, *Mexerion* is a rosette that grows appressed to the ground in the shape of a flat circle, making other size measurements such as height uninformative. Second, because we were tracking plant longevity, we could not use other traits that required an invasive measurement’

Plant size does in fact refer to plant diameter. We have amended our description in the M&M section, it now reads as (L184-5): ‘Plant diameter (hereafter referred to as plant size) and survival were measured at the beginning of the experiments.’

**Comment 2.** Reconsider what traits are included in the main paper figures vs. the supplemental. And I suggest moving the data on actual temperature treatments and hydric treatments achieved (results showed as Figure 1 and 2) to the methods section where it is first described. It’s just experimental treatment effects but not experiment results (except for the control with or without tussocks, which can be the experiment results).

*Answer:*

We changed the data about temperature and hydric treatments and figure 1 to methods section (L222-236, 247-250). Figure 2 was sent to the appendix on soil moisture.

**Comment 3**. “Five stressors in the field” (in the Abstract) and “plants were randomly assigned to eight treatments designed” (in the M&M section). Different expressions can easily mislead the reader.

*Answer:* We have changed the wording in the abstract to clarify our statement. It now reads as (L24): ‘We employed eight experimental treatments to manipulate five stressors in the field’.

**Comment 4.** In the discussion section, authors need to focus on the specific effect of different single stressors (UV- vs UV+, MIN vs EXP, MIN vs EXP, SOI vs EXP, HYD vs EXP) and the comprehensive effects of tussocks (TUS vs EXP) on plant performance.

*Answer:* We agree with the point, the past version of the manuscript did not include a thorough comparison of individual treatments. We have now added a paragraph were these comparisons are included. Following this new paragraph, we maintained the previous structure of the discussion, focusing on particular stressors. The new paragraph reads as (L311-321): “We found that plants under most treatments did not live as long as those in TUS, with those in EXP having the lowest level of life expectancy, highlighting the role of tussocks as facilitators. Factors related to the soil (HYD and SOI) had comparably low levels of life expectancy to EXP, indicating that they do not play an important role in determining life expectancy. The same can be concluded for UV radiation as plants in UV+ and UV- did not differ from each other in terms of both life expectancy and growth. However, it seems that increased water content in the soil promoted growth, suggesting that facilitation by tussocks may in part be due to water stress amelioration. The only treatments that caused a significant increase in life expectancy were those related with temperature, and MIN was the only treatment were plants lived as long as those in TUS. This indicates the important role that extreme temperatures play in facilitation by Festuca.”

**Comment** Line 43: soils are frequently sandy in high-altitude environments? I don’t think so expanding to the globe.

*Answer:* True, soils may not be sandy in other high-altitude environments. We have rephrased the sentence to clarify that sandy soils are expected in high-altitude volcanic environments. It now reads as (L43): ‘High-altitude environments are harsh. UV radiation is very intense due to the comparatively thin atmospheric layer above mountains [1, 2]. In volcanoes, soils are frequently sandy…’

**Comment** Lines 77-78: should be carefully.

*Answer:* We have attenuated our claim. It now reads as (L78): ‘Given the large variations in temperature in páramos [3, 5], it seems likely that the buffering of maximum and minimum temperatures is an important driver of facilitation by *Festuca*’.

**Comment** Line 90: 5.5 oC

*Answer:* We have fixed our spelling mistake. (L90): 5.5 °C.

**Comment** Lines 100-102: I suggest to delete this sentence.

*Answer:* We agree, the sentence did not bear particular relevance. We have deleted it.

**Comment** Line 127: where did the soil in treatment EXP come from?

*Answer:* Soil for the EXP treatment came directly from the study site. Plants we directly planted in the study site in areas were no vegetation was present. We have rephrased our sentence to clarify this point. It now reads as (L126-7): ‘Fully exposed treatment (EXP): As in the remainder of the treatments, plants were placed in areas without vegetation present in a radius of at least 1.5 m. The soil used was that of the study site where no other vegetation occurred in the vicinity.’

**Comment** Line 186: “in four subsequent occasions” need more details.

*Answer:* We have now expanded our description of when we visited the experimental site to record measurements. It now read as (L190-193): ‘Plant diameter and survival were measured at the beginning of the experiments. We visited the experimental site four times after the start of the experiments (at days 20, 93, 165 and 201 after plant transplant) to record plant diameter and survival. Six months after the start of the experiments, when only four *Mexerion* individuals were still alive, we recorded the final size and survival measurements.’

**Comment** Line 292-293: why it is the most important factor?

*Answer:* Our temperature measurements in bare ground reveal very high (> 60 °C) and very low (< 0°C) temperatures to occur in our study site. Thus, we conclude that out of the environmental factors we screened, the most important individual one driving facilitation was extreme temperatures. We have reworded our sentence, it now reads as (L301-2): ‘However, given the extreme temperatures that we recorded in the field and the positive effect of temperature-ameliorating treatments, the regulation of extreme temperatures seems to be the most important factor.’

**Comment** Appendix data: the last one is sheet “Max temperature” in the data description.

*Answer:* We have corrected the data description in our appendix data file. It now correctly refers to the ‘Max temperature’ sheet.

**REVIEWER 2**

**Comment 1**. The title should directly show the main conclusion.

*Answer:* We have updated our title. It now read as: ‘Tussocks facilitate their neighbours mainly by ameliorating extreme temperatures in tropical high mountains.’

**Comment 2.** In Fig.1 and Fig.3 legend. 'See text for treatment abbreviations.' Should list the full name of each treatment.

*Answer:* We have included the full name of each treatment for both figures 1 and 3.
