# Supplementary material for: Tussocks facilitate their neighbours mainly by ameliorating extreme temperatures in tropical high mountains
Source: PLoS One. 2020 Nov 13;15(11):e0242313. doi: 10.1371/journal.pone.0242313 (PMC7665801; doi:10.1371/journal.pone.0242313)
Supplement: Attachment — Submitted filename: Response_to_reviewer_comments_F.docx [file pone.0242313.s004.docx]

RESPONSE TO REVIEWER COMMENTS

(Line numbers correspond to the document without change control)

**REVIEWER 1**

**Comment 1:** The hypothesis posited to be a little naive in that it is very likely that all five factors are going to have an effect on the plant performance. i'd like a bolder hypothesis, based (obviously) on theory, that which factor (or factors) will be more preponderant than the others.

*Answer:* We agree with the comment, the hypothesis was in fact too vague. We have rewritten it to highlight the expected role of extreme temperature buffering in driving facilitation in páramos. It now reads as (L74-76): ‘Given the large variations in temperature in páramos [3, 5], we hypothesise the buffering of maximum and minimum temperatures to be a crucial driver of facilitation by *Festuca*.’

**Comment 2, Line 185**: how did author measure plant survival at the beginning of the experiments? And the author need to indicate that the measure of survival is for the measure of longevity.

*Answer:* Our description of survival and longevity was not clear. We have modified the sentence which read ‘We measured plant diameter (hereafter referred to as plant size) and survival were measured at the beginning of the experiments’. It now reads as (L188-189): ‘We measured plant diameter (hereafter referred to as plant size) at the beginning of the experiments’. Furthermore, to make clear how we used the survival data, we added the following sentence (L196-197): We determined the longevity of all *Mexerion* individuals from the survival data recorded in the field.

**Comment 3, Lines 185-190**: the second reason is strained. For example, you can harvest the plant for biomass at the end of the experiment. So I suggests to delete it.

*Answer:* We have deleted the sentence. The string of sentences now read as (188-193): ‘We measured plant diameter (hereafter referred to as plant size) at the beginning of the experiments. Though many plant traits can be used as surrogates for performance (e.g. plant height, root growth or leaf traits), we chose to only measure diameter and longevity. Since *Mexerion* is a rosette that grows appressed to the ground in the shape of a flat circle, other size measurements such as height would have been uninformative.’
